# Simultaneous Determination and Pharmacokinetic Comparisons of Multi-Ingredients after Oral Administration of *Radix Salviae Miltiorrhizae* Extract, Hawthorn Extract, and a Combination of Both Extracts to Rats

**DOI:** 10.1155/2014/617367

**Published:** 2014-02-10

**Authors:** Yu-Qiang Liu, Qian Cai, Chang Liu, Feng-Wei Bao, Zhen-Qiu Zhang

**Affiliations:** School of Medicine, Liaoning University of Traditional Chinese Medicine, Dalian 116600, China

## Abstract

A simple and sensitive HPLC method was developed for simultaneous determination of danshensu (DSS), rosmarinic acid (RA), lithospermic acid (LA), salvianolic acid B (SAB), and hyperoside (HP) in rat plasma. This method validated was successfully applied to the pharmacokinetic study of the main active ingredients after oral administration of *Radix Salviae Miltiorrhizae *extract (SME), hawthorn extract (HTE), and a combination of both extracts (2.5 : 1) to rats. The results indicated that there have been great differences in pharmacokinetics between a single extract and a combination of both extracts. A combination of both extracts can enhance their bioavailabilities and delay the elimination of SAB and DSS in rats.

## 1. Introduction

*Radix Salviae Miltiorrhizae* (root of *Salvia miltiorrhiza* Bge., commonly known as “Danshen”) has the functions of invigorating blood, removing stagnation, cooling blood, reducing carbuncles, clearing heat in the heart, and soothing irritability [1, 2]. It has been used widely in traditional Chinese medicine to treat various cardiovascular diseases. Recent researches show that the main active ingredients are the hydrosoluble components called salvianolic acids, containing salvianolic acid B, protocatechuic aldehyde (PA), danshensu, rosmarinic acid, and so on [3]. Pharmacological studies show that the salvianolic acids can protect the myocardium from ischemia-induced derangement, protect neural cells from injuries caused by anoxia, inhibit platelet aggregation, reduce hepatic fibrosis, and depress the activities of HIV-1 [4, 5].

Hawthorn (*Crataegus pinnatifida *Bge.var.*major *N.E.Br. or *Crataegus pinnatifida* Bge.) has been an edible and medicinal fruit in traditional Chinese medicine with the functions of promoting digestion and invigorating the stomach, eliminating stasis to activate blood circulation, and lowering blood lipid, so often used for treating diseases of cardiovascular system and alimentary system. The main active ingredients are organic acids and flavonoids, containing hyperin, kaempferol and quercetin, and so forth. Recently, pharmacological studies have shown that the total flavonoids possess a wide range of pharmacological properties, such as improving coronary and cerebral blood flow, reducing blood lipid, protecting against arrhythmias, increasing cardiac activity, and inhibiting platelet aggregation [6, 7].

**Figure 1:**
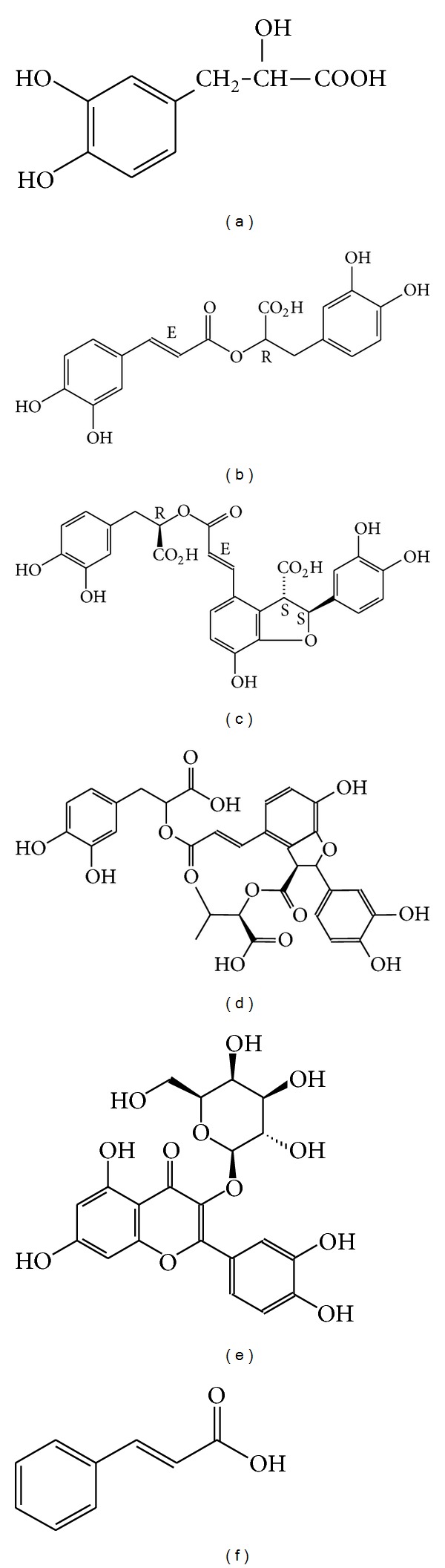
Chemical structures of DSS (a), RA (b), LA (c), SAB (d), HP (e), and CA (f).

In clinical practice, Hawthorn and *Radix Salviae Miltiorrhizae* are commonly used herbs for reducing blood lipid and are compatible with each other. The primary experiments of pharmacology confirmed that a combination of SME and HTE (2.5 : 1) had more obvious effects in terms of lipid-decreasing, improving blood rheological characteristic, inhibiting platelet aggregation, antimyocardial oxygen consumption, and so forth, in comparison with a single SME or HTE [8]. Until now, no reports have studied the determination of multi-ingredients in biological samples and their pharmacokinetics after an oral administration of a combination of both extracts. Therefore, it is necessary to investigate the pharmacokinetics of a combination of both extracts. In this study, a simple and sensitive HPLC method validated was developed for simultaneous determination of DSS, RA, LA, SAB, and HP in rat plasma, and the concentrations of the five ingredients in rats plasma were determined by this method after SME, THE, and a combination of both extracts (2.5 : 1) were orally given to rats, respectively. The purpose of this study was to investigate the difference in the pharmacokinetics after oral administration of a single extract (SME or HTE) and a combination of both extracts to rats. Meanwhile, it will provide a theoretical basis for a combination of both extracts by comparing the pharmacokinetic parameters of the five ingredients. The chemical structures of the DSS, RA, LA, SAB, HP, and CA were shown in Figure 1.

## 2. Experimental

### 2.1. Reagents and Chemicals

DSS, RA, LA, SAB, CA (IS), and HP standards (the purities of them >98%) were all obtained from National Institute for Food and Drug Control (Beijing, China). The traditional Chinese medicine of *Radix Salviae Miltiorrhizae* and hawthorn were purchased from Hebei Anguo Pharmacy Group (China). The material was authenticated by professor Feng Li from Liaoning University of Traditional Chinese Medicine. HPLC grade acetonitrile and analytical grade ethyl acetate, phosphoric acid, perchloric acid, and other reagents were obtained from Tianjin Kemiou Chemical Reagent Co., Ltd. (China). Pure water (Hangzhou Wahaha Group Co., Ltd., China) was filtered through 0.22 mm filter membrane before use.

### 2.2. Preparation of the SME and HTE

The preparation of SME: *Radix Salviae Miltiorrhizae* was extracted three times (2.0 h for each time) with water. Then the extract was filtered and evaporated to the concentration of 1 mL equal to 0.2 g crude drug. The concentrated solution was chromatographed on AB-8 macroporous resin (Cangzhou Bon Adsorber Technology Co., Ltd., Hebei, China). The chromatography column was washed first with water, and then desorbed with 50% ethanol. The eluent was concentrated in the rotary evaporation apparatus and dried under vacuum at 60°C. The contents of DDS, RA, LA, and SAB in the extract were detected by HPLC, 1.2, 2.6, 5.9, and 48.0 mg/g, respectively.

The preparation of HTE: hawthorn was extracted two times (2.0 h for each time) with 75% ethanol, then the extract was filtered and evaporated to the concentration of 1 mL equal to 1.0 g crude drug by rotary evaporation at 60°C under vacuum. The concentrated solution was chromatographed on AB-8 macroporous resin. The chromatography column was washed first with water, and then desorbed with 70% ethanol. The eluent was concentrated in the rotary evaporation apparatus and dried under vacuum. The content of HP in the extract was detected by HPLC, 3 mg/g.

### 2.3. Animals

Male, Sprague-Dawley rats (200 ± 20 g) were obtained from the Experimental Animal Center of Dalian Pharmaceutical University and kept in environmentally controlled breeding room for one week before starting the experiments.

### 2.4. Liquid Chromatographic Condition

The liquid chromatography system employed was Agilent 1100 with variable wavelength UV detector (G1314A VWD). Data were analyzed by Agilent ChemStation (Agilent Technologies Inc., China). Separation was achieved on a column (250 × 4.6 mm, 5 *μ*m, Dikma Technologies, China) filled with ODS and a C_18_ guard column (10 × 4.6 mm, 5 *μ*m, Welch Materials Inc., China). The mobile phase consisted of 0.1% phosphoric acid solution (A) -acetonitrile (B) with gradient elution of 94–83% A at 0–13 min; 83–79% A at 13–38 min; and 79–69% A at 38–55 min. The wavelength-switching technology was performed to determine DSS at 280 nm (0–20 min), HP at 257 nm (20–38 min), and RA, LA, and SAB at 289 nm (38–55 min). The flow rate was 1.0 mL/min. An injection volume was 20 *μ*L.

### 2.5. Preparation of Standard Solutions and Quality Control Samples

The stock solutions of DSS, RA, LA, SAB, and HP with concentrations of 647, 604, 703, 748, and 600 *μ*g/mL, respectively, were all prepared in methanol. The concentration of the IS solution dissolved in methanol was 50 *μ*g/mL. These stock solutions were diluted to serial standard working solutions with methanol at concentrations of 3.235, 6.47, 16.175, 32.35, 64.7, and 161.75 *μ*g/mL for DSS; 3, 6, 15, 30, 60, and 150 *μ*g/mL for HP; 3.02, 6.04, 15.1, 30.2, 60.4, and 151 *μ*g/mL for RA; 3.515, 7.03, 17.575, 35.15, 70.3, 175.75, and 351.5 *μ*g/mL for LA; 3.74, 7.48, 18.7, 37.4, 74.8, 187, and 374 *μ*g/mL for SAB. The solutions mentioned above were then added to blank plasma (1 : 20) to make series standard solutions of 0.162–8.088 *μ*g/mL for DSS, 0.15–7.50 *μ*g/mL for HP, 0.151–7.55 *μ*g/mL for RA, 0.175–17.575 *μ*g/mL for LA, and 0.187–18.7 *μ*g/mL for SAB. The quality control (QC) samples used for the inter- and intraday precision, accuracy, stability, and the extraction recovery were prepared in the same way.

### 2.6. Sample Preparations

The samples of rat plasma (100 *μ*L) were mixed with 20 *μ*L IS working solution and 50 *μ*L perchloric acid (6%, v/v). After protein was precipitated in a 1.5 mL polypropylene tube by vortexing for 3 min, the samples were centrifuged at 4,280 g for 5 min. The supernatant transferred into a new clean tube was extracted twice with 500 *μ*L ethyl acetate. The ethyl acetate layer was evaporated to dryness under the stream of nitrogen. The residue was dissolved with 50 *μ*L methanol and then centrifuged at 4,280 g for 3 min. The 20 *μ*L of the supernatant was injected for analysis.

### 2.7. Method Validation

The method was fully validated for its specificity, lower limit of detection (LOD), lower limit of quantification (LOQ), linearity, precision, and accuracy. The intra- and inter-day precision and accuracy were evaluated by assaying the QC samples with high, medium, and low concentrations that could be quantified with an accuracy between 80% and 120%. The extraction recovery from plasma was determined at three concentrations by comparing the peak areas of plasma extracts with those of the same amounts of standard solutions. The extraction recovery of IS (CA) from plasma was also determined by the same method. Three concentrations of plasma samples spiked with DSS, RA, LA, SAB, and HP, respectively, were determined at room temperature for 8 h as well as storage at −20°C for 30 days to validate their stabilities.

### 2.8. Applications in Pharmacokinetic Study

#### 2.8.1. Sample Collection

The rats were made to fast for 12 h but given free access to water before the extract solution was orally administrated at a dose of SME 6 g/kg, HTE 2.4 g/kg, and a combination of both extracts 8.4 g/kg (2.5 : 1), respectively. Orbital venous blood samples (0.5 mL) were collected before dosing, and at 0.08, 0.17, 0.33, 0.5, 0.75, 1.0, 1.5, 2, 3, 4, 6, 8, and 12 h after an administration. Then the plasma samples obtained were immediately stored at −20°C until analysis after centrifuging at 4,280 g for 5 min.

#### 2.8.2. Pharmacokinetic Analysis

HPLC analysis procedure was applied to analyze plasma concentration-time profiles of DSS, RA, LA, SAB, and HP. Data were processed by noncompartmental method using Drug and Statistics (DAS) 2.0 software package (Chinese Pharmacological Society, Shanghai, China).

## 3. Results

### 3.1. Selection of Wavelength

The liquid chromatography with CA as the internal standard was carried out with gradient elution and wavelength switching. DSS was found the maximum absorption wavelength at 280 nm, IS at 273 nm, HP at 257 and 360 nm, RA at 290 and 328 nm, LA at 254, 289, and 310 nm and SAB at 254, 288, and 307 nm, respectively. In order to obtain the high sensitivities for every component and reduce the baseline shift, finally, the different absorption wavelengths were set at 280 nm for DSS, 257 nm for IS and HP, and 289 nm for RA, LA, and SAB, respectively. Under the chromatographic conditions described above, the respective retention times of DSS, HP, RA, LA, SAB, and IS were suitable and both endogenous and metabolic components in rat plasma showed no interference.

### 3.2. Method Validation

#### 3.2.1. Specificity and Linearity

**Figure 2:**
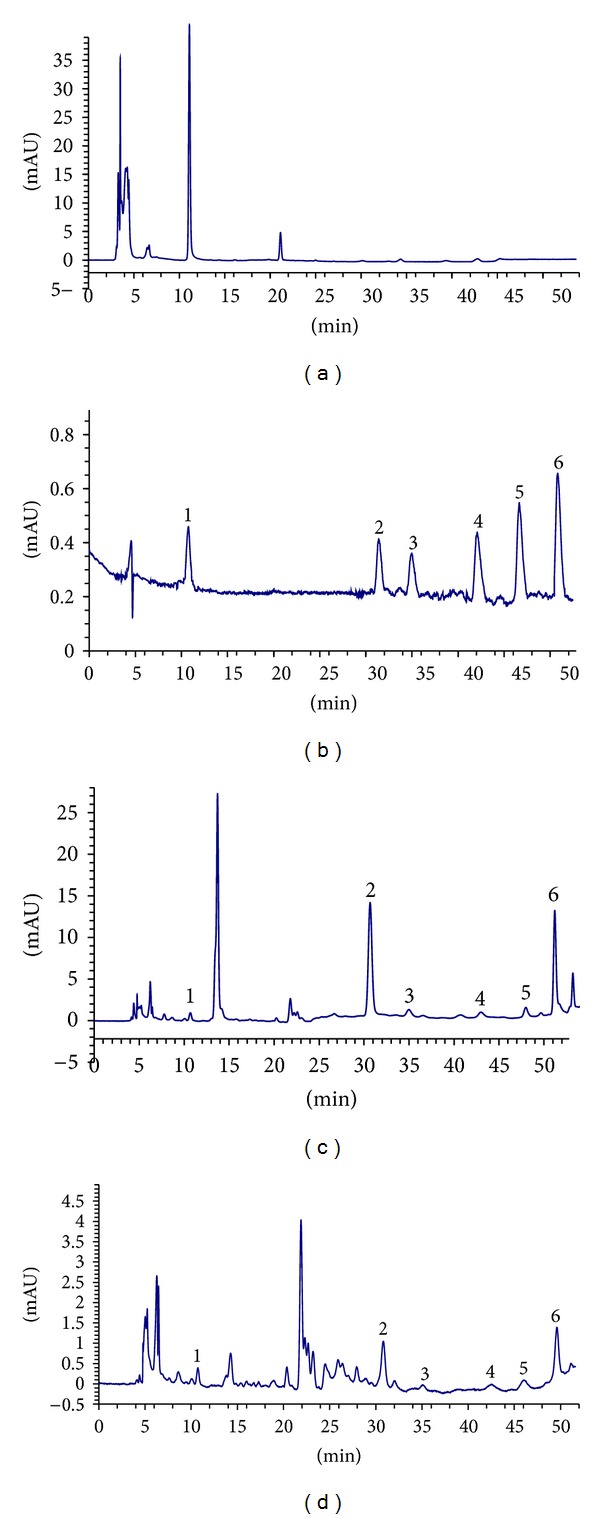
Typical HPLC chromatograms of the analytes in rats plasma. (a) Chromatogram of blank plasma; (b) chromatogram of standards; (c) blank plasma spiked with DSS, RA, LA, SAB, HP, and IS; and (d) chromatogram of a plasma sample at 10 min after oral administration of a combination of both extracts. Peak 1: DSS; peak 2: HP; peak 3: IS; peak 4: RA; peak 5: LA; and peak 6: SAB.

The typical HPLC chromatograms were shown in Figure 2. Under the chromatographic conditions described above, DSS, IS, HP, RA, LA, and SAB were eluted with retention times of 10.6, 30.4, 34.7, 42.2, 47.8, and 51.2 min, respectively. Endogenous components in rat plasma did not show any interfering peaks. Suitable amounts of DSS, RA, LA, SAB, and HP were weighed and dissolved in methanol as a stock solution. Each solution was diluted to a suitable concentration, and a linear regression of the standard concentration versus peak area ratios by weighted (1/*x* ^2^) linear least-squares regression method was plotted. The results of the calibration curves and the lower limit of detections (LODs) of the five components were shown in Table 1.

#### 3.2.2. Precision and Accuracy

Three concentrations of DSS (8.10, 1.62, and 0.324 *μ*g/mL), RA (7.55, 1.51, and 0.302 *μ*g/mL), LA (17.6, 3.52, and 0.879 *μ*g/mL), SAB (18.7, 3.74, and 0.935 *μ*g/mL), and HP (7.5, 1.5, and 0.3 *μ*g/mL) standard stock solutions added to blank plasma samples, respectively, were determined five times on the same day to measure the intra-day accuracy and for three consecutive days to measure the inter-day accuracy. The precision and accuracy data were shown in Table 2. The intra- and inter-day RSD values were lower than 10%. The results showed satisfactory precision and accuracy of this present method.

#### 3.2.3. Recovery

The extraction recovery was evaluated by comparing the peak areas of the extracted QC samples at three concentrations (high, medium, and low) with those of the unextracted standards that represented 100% recovery. Similarly, the recovery of IS was evaluated at a single concentration by the same method. The results showed that the extraction method was suitable to extract SAB, RA, LA, DSS, HP, and IS from plasma. The recoveries of the five components and IS were stable. The validated HPLC method was proved to be successfully applied to the pharmacokinetic study after an oral administration of SME, THE, and a combination of both extracts to rats. The recoveries at each concentration were shown in Table 3.

#### 3.2.4. Stability

The stabilities of SAB, RA, LA, DSS, and HP in rat plasma were evaluated by analyzing the stored QC samples at three concentrations. The QC samples were kept at room temperature for 12 h and then determined. Long-term stability was assessed by keeping QC samples at –20°C for 30 days. The freeze-thaw stability was determined after three freeze (–20°C, 24 h) and thaw (room temperature) cycles. The results indicated that the five active components showed sufficient stability under the respective conditions. The results were shown in Table 4.

### 3.3. Pharmacokinetic Study

**Figure 3:**
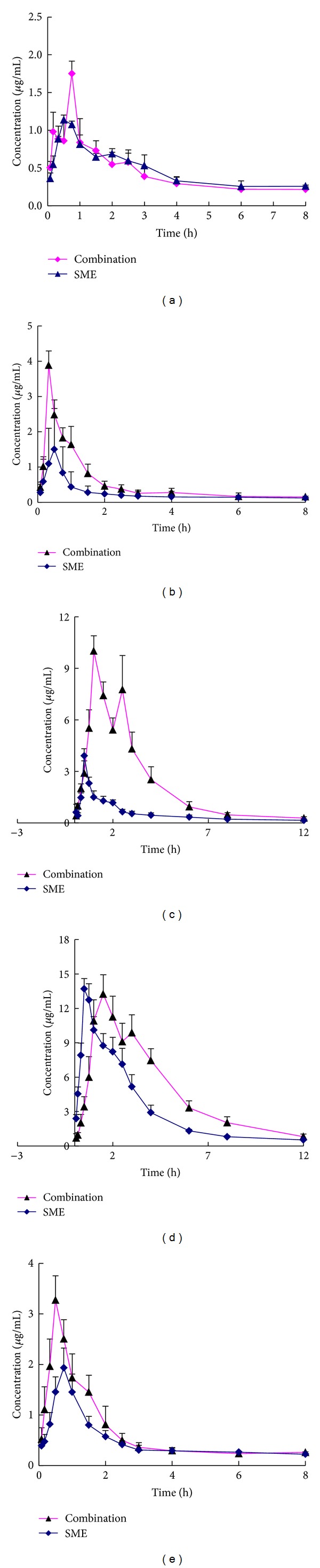
Mean concentration-time profiles of DSS (a), RA (b), LA (c), SAB (d), and HP (e) in rat plasma after oral administration of a single extract and a combination of both extracts.

The mean plasma concentration-time profiles of DSS, RA, LA, SAB, and HP were illustrated in Figure 3 and the pharmacokinetic parameters were shown in Table 5. By comparing the pharmacokinetic data of the SME, THE, and a combination of both extracts with *t*-test, the results can be concluded that there have been significant differences between them.

## 4. Discussions

Salvianolic acids, such as DSS, RA, LA, and SAB are the main active ingredients of *Salvia miltiorrhiza* Bunge. Flavonoids, such as HP, are the major active ingredients of Hawthorn. Some reports about the pharmacokinetics of the single SME have been published [9–13], but so far there has been still no information about the simultaneous determination and pharmacokinetic studies after administration of a combination of both SME and HTE.

To select the IS, the compounds such as benzoic acid, *p*-hydroxy benzoic acid, and baicalin [14, 15] mentioned in some reports about SME have been interfered because of the extracts and metabolite; cinnamic acid was finally chosen as the IS for the assay.

In comparison with the concentration-time profiles of every ingredient after oral administration of a single extract and a combination of both extracts to rats, all of the five ingredients have a short *T*_max⁡_ and can be quickly absorbed *in vivo* after oral administration of a single extract, while the *T*_max⁡_ of RA and HP was shorter after administration of a combination of both extracts. This may mean a combination of both extracts can quicken the absorption of RA and HP. From the pharmacokinetic parameters with the noncompartmental method, it was found that the AUCs of DSS, RA, LA, SAB, and HP after an oral administration of a combination of both extracts were increased, which were 4.44, 4.25, 27.3, 56.5, and 5.24 mg·h/L, respectively, while the AUCs of the five ingredients after an oral administration of a single extract were 3.65, 2.09, 6.49, 38.2, and 3.84 mg·h/L, respectively. Among the five ingredients, the AUCs of RA, LA, and SAB were increased significantly (*P* < 0.01). The *C*_max⁡_ of above five ingredients after an oral administration of a combination of both extracts was 1.6, 2.6, 2.6, 1, and 1.7 times compared to that after an oral administration of single extract, which showed that a combination of both extracts can enhance the absorption of DSS, RA, LA, and HP, but the *t*_1/2_ and MRT of RA, LA, and HP were decreased after an oral administration of a combination of both extracts, which indicated that the absorption was increased and the elimination was quickened. The *t*_1/2_ of SAB was increased and its *C*_max⁡_ was not changed after an oral administration of a combination of both extracts, which showed that its AUC was increased due to the slowing of its elimination. The interactions of multi-ingredients in a combination of both extracts may be the reason of above different pharmacokinetic parameters.

In this study, the pharmacokinetic parameters after an oral administration of a single extract and a combination of both extracts were compared with DSS, RA, LA, SAB, and HP as indexes, and the results showed that a combination of both extracts can cause great changes in the pharmacokinetics and this might be helpful as guide when using a combination of traditional Chinese medicine.

## 5. Conclusion

A simple and sensitive HPLC method was developed for simultaneous determination of DSS, RA, LA, SAB, and HP in rat plasma. This method validated was successfully applied to the pharmacokinetic study of the main active ingredients after oral administration of SME, THE, and a combination of both extracts. The results indicated that there have been great differences in pharmacokinetics between a single extract and a combination of both extracts.

## Figures and Tables

**Table 1:** Calibration curves of the five ingredients.

Analyte	LOD (*μ*g/mL)	Slope ± SD	Intercept ± SD	*r*	Linear range (*μ*g/mL)
DSS	0.04	0.1134 ± 0.005	−0.0347 ± 0.0017	0.9936	0.16–8.0
RA	0.05	0.1909 ± 0.012	−0.0031 ± 0.0003	0.9938	0.151–7.55
LA	0.04	0.1813 ± 0.011	−0.0437 ± 0.0035	0.9932	0.175–17.5
SAB	0.06	0.2604 ± 0.013	0.0964 ± 0.0012	0.9919	0.187–18.7
HP	0.05	0.0926 ± 0.006	−0.0095 ± 0.0007	0.9972	0.15–7.5

**Table 2:** Intra- and interday precision and accuracy for QC samples (*n* = 5).

Aanlyte	Added (*μ*g/mL)	Interday			Intraday		
		Found (*μ*g/mL)	RSD (%)	Accuracy (%)	Found (*μ*g/mL)	RSD (%)	Accuracy (%)
DSS	8.10	7.88 ± 0.41	5.18	97.5	8.08 ± 0.46	5.75	99.9
	1.62	1.61 ± 0.07	4.43	100.1	1.61 ± 0.09	5.90	99.0
	0.324	0.328 ± 0.02	6.44	101.2	0.325 ± 0.03	7.73	100.4

HP	7.50	7.65 ± 0.33	4.25	102.0	7.82 ± 0.16	2.02	104.2
	1.50	1.52 ± 0.08	5.05	101.2	1.51 ± 0.08	5.45	100.5
	0.300	0.307 ± 0.02	6.16	102.4	0.315 ± 0.01	4.52	104.9

RA	7.55	7.67 ± 0.29	3.78	101.5	7.90 ± 0.10	1.26	102.7
	1.51	1.49 ± 0.07	4.44	98.8	1.51 ± 0.07	4.76	100.3
	0.302	0.307 ± 0.02	5.62	101.6	0.299 ± 0.02	6.57	99.2

LA	17.6	17.8 ± 0.65	3.64	101.1	17.7 ± 0.69	3.91	100.7
	3.52	3.58 ± 0.15	4.10	101.8	3.57 ± 0.12	3.41	101.7
	0.879	0.865 ± 0.04	4.58	98.4	0.885 ± 0.03	3.80	100.7

SAB	18.7	18.5 ± 0.78	4.19	98.9	18.9 ± 0.72	3.79	101.0
	3.74	3.64 ± 0.14	3.81	97.3	3.65 ± 0.16	4.32	97.5
	0.935	0.932 ± 0.05	4.92	99.7	0.927 ± 0.07	7.40	99.2

**Table 3:** Recoveries of SAB, RA, LA, DSS, and HP (*n* = 3).

Aanlyte	Added (*μ*g/mL)	Found (*μ*g/mL)	Recovery (%)	Aanlyte	Added (*μ*g/mL)	Found (*μ*g/mL)	Recovery (%)
DSS	8.10	5.83 ± 0.13	72.1	HP	7.50	5.33 ± 0.17	71.1
	1.61	1.21 ± 0.03	74.5		1.50	1.12 ± 0.05	74.7
	0.324	0.232 ± 0.02	71.6		0.300	0.207 ± 0.01	69.1

RA	7.55	5.94 ± 0.16	78.7	LA	17.6	13.4 ± 0.32	76.5
	1.51	1.15 ± 0.06	76.3		3.52	2.73 ± 0.11	77.8
	0.302	0.22 ± 0.01	74.3		0.879	0.638 ± 0.04	72.6

SAB	18.7	15.2 ± 0.38	81.3	IS	10.0	7.64 ± 0.29	76.4
	3.74	2.96 ± 0.09	79.2				
	0.935	0.697 ± 0.02	74.6				

**Table 4:** Stabilities of SAB, RA, LA, DSS, and HP (*n* = 3).

Aanlyte	Added (*μ*g/mL)	Stored at room temperature for 12 h		Stored at −20°C for 30 days		Three freeze-thaw stability	
		Found (*μ*g/mL)	RSD (%)	Found (*μ*g/mL)	RSD (%)	Found (*μ*g/mL)	RSD (%)
DSS	8.10	7.94 ± 0.18	2.22	7.63 ± 0.31	4.00	7.72 ± 0.06	0.80
	1.62	1.57 ± 0.04	2.55	1.56 ± 0.07	4.40	1.48 ± 0.06	4.08
	0.324	0.315 ± 0.02	4.77	0.313 ± 0.03	9.51	0.308 ± 0.02	6.02

HP	7.50	7.30 ± 0.11	1.47	7.02 ± 0.24	3.47	6.99 ± 0.25	3.53
	1.50	1.48 ± 0.02	1.02	1.37 ± 0.03	2.40	1.41 ± 0.05	3.37
	0.300	0.288 ± 0.02	4.83	0.280 ± 0.02	7.72	0.290 ± 0.02	7.73

RA	7.55	7.37 ± 0.19	2.52	7.18 ± 0.26	3.65	7.16 ± 0.36	5.05
	1.51	1.50 ± 0.05	3.08	1.41 ± 0.04	2.52	1.40 ± 0.03	2.33
	0.302	0.292 ± 0.02	4.95	0.275 ± 0.02	7.94	0.286 ± 0.03	8.93

LA	17.6	17.1 ± 0.16	0.96	16.4 ± 0.55	3.34	16.2 ± 0.39	2.38
	3.52	3.39 ± 0.06	1.63	3.16 ± 0.21	6.48	3.17 ± 0.17	5.33
	0.879	0.797 ± 0.03	3.24	0.773 ± 0.06	7.40	0.785 ± 0.04	4.45

SAB	18.7	18.2 ± 0.42	2.29	17.7 ± 0.75	4.27	17.6 ± 0.38	2.17
	3.74	3.63 ± 0.13	3.46	3.44 ± 0.13	3.65	3.55 ± 0.14	3.93
	0.935	0.894 ± 0.05	5.36	0.861 ± 0.02	2.51	0.851 ± 0.05	5.72

**Table 5:** Pharmacokinetic parameters of DSS, RA, LA, SAB, and HP in rat plasma after oral administration of a single extract and a combination of both extracts (*n* = 6).

Parameter	DSS	DSS′	RA	RA′	LA	LA′	SAB	SAB′	HP	HP′
AUC_0–t_ (mg·h/L)	3.65 ± 0.42	4.44 ± 0.66*	2.09 ± 0.39	4.25 ± 0.78**	6.49 ± 0.88	27.3 ± 3.0**	38.2 ± 3.0	56.5 ± 6.52**	3.84 ± 0.61	5.24 ± 0.79*
AUC_0–∞_ (mg·h/L)	4.29 ± 0.99	5.22 ± 3.7	3.39 ± 2.3	5.20 ± 1.8	7.05 ± 2.1	27.5 ± 2.8**	38.7 ± 4.1	59.4 ± 7.64**	5.99 ± 1.0	6.52 ± 1.2
MRT_0–t_ (h)	2.98 ± 0.15	2.76 ± 0.10*	2.50 ± 0.44	1.98 ± 0.12*	3.27 ± 0.50	2.93 ± 0.22	2.85 ± 0.13	3.78 ± 0.19**	2.68 ± 0.16	2.22 ± 0.20**
MRT_0–∞_ (h)	5.52 ± 1.1	5.26 ± 0.75	8.86 ± 5.4	4.24 ± 10.0	5.06 ± 3.9	3.24 ± 0.29*	3.38 ± 1.1	4.36 ± 0.36	9.04 ± 1.1	6.80 ± 3.3*
*T*_(1/2)z_ (h)	2.85 ± 1.2	3.00 ± 8.8	7.52 ± 4.4	4.34 ± 9.0*	3.78 ± 3.8	1.60 ± 0.67*	1.94 ± 2.1	2.50 ± 0.45	7.00 ± 0.67	5.35 ± 3.0
*T*_max⁡_ (h)	0.5	0.75	0.5	0.33	0.5	1.0	0.5	1.5	0.75	0.5
*C*_max⁡_ (mg/L)	1.14 ± 5.8	1.87 ± 0.17**	1.50 ± 0.44	3.88 ± 0.41**	3.91 ± 0.41	10.0 ± 0.89**	13.7 ± 0.88	13.2 ± 1.7	1.93 ± 0.39	3.27 ± 0.48**

′A combination of both extracts. *P < 0.05; **P < 0.01; comparison with a single extract (SME or HTE).
